# A Diol-Based-Matrix Solid-Phase Dispersion Method for the Simultaneous Extraction and Determination of 13 Compounds From *Angelicae Pubescentis Radix* by Ultra High-Performance Liquid Chromatography

**DOI:** 10.3389/fphar.2019.00227

**Published:** 2019-03-08

**Authors:** Mingya Ding, Yun Bai, Jin Li, Xuejing Yang, Hui Wang, Xiumei Gao, Yan-xu Chang

**Affiliations:** ^1^Tianjin State Key Laboratory of Modern Chinese Medicine, Tianjin University of Traditional Chinese Medicine, Tianjin, China; ^2^Tianjin Key Laboratory of Phytochemistry and Pharmaceutical Analysis, Tianjin University of Traditional Chinese Medicine, Tianjin, China; ^3^School of Pharmacy, Harbin University of Commerce, Harbin, China

**Keywords:** *Angelicae Pubescentis Radix*, coumarins, Diol-matrix solid-phase dispersion, phenolic acids, UHPLC

## Abstract

A simple and eco-friendly Diol-based-matrix solid-phase dispersion method (MSPD) was optimized and established to simultaneously extract 13 bioactive compounds (7 coumarins and 6 phenolic acids) in *Angelicae Pubescentis Radix* (APR) by ultrahigh performance liquid chromatography coupled with photodiode array detector (UHPLC-PDA). Diol was chosen as the dispersing sorbent and methanol solution was used as the elution solvent. The preparation procedures for the MSPD including the types of sorbents, mass ratio of matrix to sorbent, grinding time, type, concentration and volume of elution solvent were investigated. Under the optimal conditions, good recoveries of the 13 target compounds were obtained in the range of 94.8–107% (RSD < 3.22%). The limits of detection (LODs) and limits of quantitation (LOQs) were in the ranges of 0.08–0.12 μg mL^-1^ and 0.16–0.24 μg mL^-1^, respectively. Compared with the traditional method, it was a green and environmentally friendly technique. The results proved that the established method was successfully applied to the extraction and determination of 13 target bioactive compounds for quality control in APR.

## Introduction

*Angelicae Pubescentis Radix* (APR), the dried roots of *Angelica pubescens* Maxim, f. biserrata Shan et Yuan, belonged to the Apiaceae family. It was named as Duhuo in Chinese as a commonly used traditional Chinese medicine (TCM) (Chinese Pharmacopoeia Commission, 2015). It was first recorded in Shennong’s Classic of Materia Medica and medically used during the North-South Dynasty period (Cao and Li, 2018). APR is wildly used to treat rheumatic disease in the clinics in China (Wang et al., 2008). Recently, many studies have revealed that APR has the anti-tumor, anti-inflammation, anti-proliferatory, anti-platelet aggregation and anthelmintic activities (Liu et al., 1998, 2010; Wang et al., 2010; Chang et al., 2013). The chemistry compositions of APR included coumarins, phenolic acids, essential oils, organic acids, and saccharides (Chen, 2014). It was reported that coumarins were the most abundant in APR (Chang et al., 2011). The coumarins have attracted extensive attention worldwide. However, other compounds covering phenolic acids also have a series of pharmacological activities such as anti-oxidant and immune regulation. Moreover, osthole and columbianadin were the quality control markers in Chinese Pharmacopoeia Commission (2015 Version), which may not be specific and meaningful (Chinese Pharmacopoeia Commission, 2015). Therefore, the complete extraction and precise analysis of coumarin and phenolic acid compounds are particularly important for quality control and physiological and pharmacological investigations.

Currently, many analytical methods for determining multiple components in APR have been developed by high-performance liquid chromatography with diode array detector (HPLC-DAD), gas chromatography-mass spectrometry (GC-MS), and high-performance liquid chromatography coupled with quadrupole time-of-flight tandem mass spectrometry (HPLC-Q-TOF/MS) (Yang et al., 2006; Ge et al., 2014; Zhang et al., 2017; Wang J. R. et al., 2018). The commonly used extraction method of APR is ultrasonic-assisted extraction (UAE), which not only need large amounts of samples and a great deal of organic solvents, but also need much extraction times. Matrix solid phase dispersion (MSPD) as a promising sample extraction technique was normally employed to extract the solid, semisolid, and highly viscous biological samples by disrupting and dispersing the solid sorbent in the sample (Barker et al., 1989; Liu et al., 2008; Vela-Soria et al., 2014; Li et al., 2017). Its unique properties is that integrating sample matrix disruption, extraction, fractionation and clean up in one single process could reduce the consumption of organic solvents and deliver cost savings (Barker et al., 1989). To our knowledge, no literatures have been reported on MSPD as an extraction method for the simultaneous extraction of coumarins and phenolic acids in APR.

It has been reported that the selection of a suitable sorbent was one of the vital procedures in the development of the MSPD (Enríquez-Gabeiras et al., 2012). The sorbent could be used to disrupt the sample architecture, and employed to disperse analytes onto a solid phase support to generate powerful sample-solvent interactions (Wang H. L. et al., 2018). The commonly sorbent in MSPD were reversed-phase material [C_18_ (end capped), C_18_-N (no end capped) and AQ C_18_ (aqua C_18_)], normal-phase materials (Florisil, NH_2_, COOH, Diol) and non-silica-based supporting material (PS, PEP, PEP-2). Diol, a high purity spherical silica-based sorbent, extracts polar analytes from non-polar solutions by hydrogen bonding. Meanwhile, it could also be used to extract non-polar compounds owing to the sufficient non-polar force from carbon chain on the bonded phase.

The present work aimed to optimize and establish a simple and eco-friendly Diol-based-matrix solid-phase dispersion method for the simultaneous extraction of 6 phenolic acids (neochlorogenic acid, chlorogenic acid, 4-dicaffeoylquinic acid, isochlorogenic acid B, isochlorogenic acid A, isochlorogenic acid C) and 7 coumarins (umbelliferae, columbianetin, columbianetin acetate, imperatorin, osthole, isoimperatorin, and columbianadin) in APR by ultrahigh performance liquid chromatography coupled with PDA detection (UHPLC-PDA). The Diol was firstly employed to extract multiple components of APR during the MSPD procedure. The potential influential parameters such as the types of sorbents, mass ratio of matrix to sorbent, grinding time, eluent type, concentration and volume of eluent were investigated for acquiring optimal extraction efficiency.

## Materials and Methods

### Chemicals and Reagents

Thirteen reference standards except for columbianadin and columbianetin acetate were obtained from Chengdu Desite Bio-Technology Co., Ltd. (Chengdu, China). The standards of columbianadin and columbianetin acetate were isolated and purified from APR extract by our laboratory, which were identified by H-NMR, IR, and HPLC-MS. The purity of all analytes were more than 98%. C_18_ (50 μm, 60 A), C_18_-N (50 μm, 60 A), AQ C_18_ (50 μm, 60 A), NH_2_ (40–60 μm, 60 A), COOH (50 μm, 60 A), Diol (50 μm, 60 A), Florisil (60–100 μm, 80 A), PS (40–60 μm, 70 A), PEP (40–60 μm, 70 A) and PEP-2 (40–60 μm, 60 A) were supplied from Agela technologies. HPLC-grade acetonitrile and methanol were both provided by Dikma Technologies Inc., United States. HPLC-grade formic acid was obtained from Tedia Company Inc. (Tedia, Fairfield, OH, United States). Other chemicals were of analytical reagent grade. Ultrapure water was supplied by a Milli-Q academic ultra-pure water system (Millipore, Milford, MA, United States). All the solutions were filtered through a 0.22 μm filter membrane before UPLC analysis.

### Plant Material

The six dried roots samples of APR were collected from the Chinese medica materia markets of Hubei, Sichuan, Anhui and Zhejiang province of China and identified by Dr. Yan-xu Chang (Tianjin University of Traditional Chinese Medicine). All samples were pulverized by a pulverizer (Zhongcheng Pharmaceutical Machinery) after dried at 60° centigrade for 24 h, then passed through a 60-mesh sieve.

### Preparation of Standard Solutions

Thirteen reference standards were individually dissolved in methanol to obtain the concentration at the concentration of 2 mg mL^-1^, respectively. The stock solution containing 50 μg mL^-1^ neochlorogenic acid, 300 μg mL^-1^ chlorogenic acid, 200 μg mL^-1^ 4-dicaffeoylquinic acid, 50 μg mL^-1^ umbelliferae, 25 μg mL^-1^ isochlorogenic acid B, 50 μg mL^-1^ isochlorogenic acid A, 50 μg mL^-1^ isochlorogenic acid C, 50 μg mL^-1^ columbianetin, 50 μg mL^-1^ columbianetin acetate, 50 μg mL^-1^ imperatorin, 50 μg mL^-1^ osthole, 50 μg mL^-1^ isoimperatorin, and 50 μg mL^-1^ columbianadin was accurately mixed up. Appropriate amounts of stock solution was diluted with methanol for calibration curve. All the related standard solutions were stored at 4°C until analysis.

### UHPLC-PDA Analysis

**FIGURE 1:**
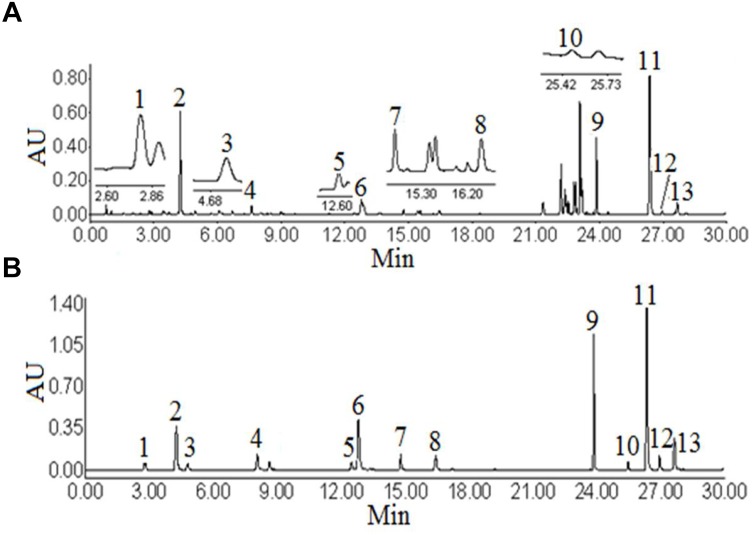
Ultra high performance liquid chromatography figure of *Angelicae Pubescentis Radix* **(A)**, mixture of standard compounds **(B)**. Peaks: 1, neochlorogenic acid; 2, chlorogenic acid; 3, 4-dicaffeoylquinic acid; 4, umbelliferae; 5, isochlorogenic acid B; 6, isochlorogenic acid A; 7, isochlorogenic acid C; 8, columbianetin; 9, columbianetin acetate; 10, imperatorin; 11, osthole; 12, isoimperatorin; 13, columbianadin.

The UHPLC-PDA analysis for this work was performed on a Waters ACQUITY UPLC System (Waters Co., Milford, MA, United States) coupled with a photodiode array detector. The workstation system was supported by Empower 2 software for data collection and analysis. The condition of the UHPLC-PDA was referred to the previous studies (Ge et al., 2014). An ACQUITY UPLC BEH C_18_ column (2.1 × 100 mm, 1.7 μm, Waters) was employed to separate the target analytes. The mobile phase consisted of water with 0.1% formic acid (eluent A) and acetonitrile (eluent B) using a gradient elution. The ratios of acetonitrile were 5, 8, 10, 14, 16, 16, 18, 20, 21, 23, 30, 50, 51, 95, 5% B at 0, 2, 5, 8, 9, 12, 13, 14, 15, 18, 21, 23, 28, 29, 30 min, respectively. The flow rate was 0.3 mL min^-1^, then post-run 6 min. The column temperature was set at 35°C. The injection volume was 2 μL. The wavelength of the photodiode array detector was set at 320 nm. Under the above chromatographic conditions, the chromatographic peaks of analytes included samples and standard solutions were separated excellently (Figure 1).

### MSPD Procedure

**FIGURE 2:**
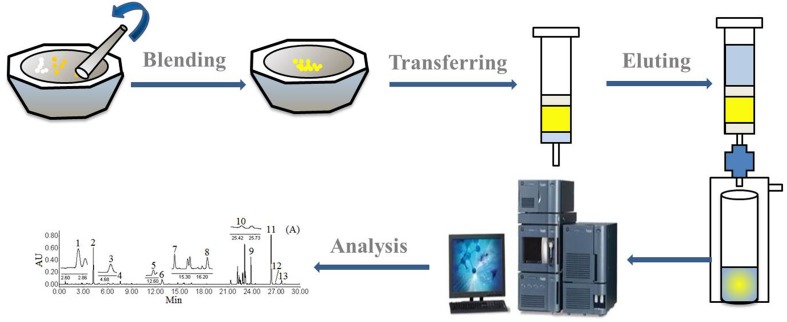
Schematic diagram of the MSPD method.

The crushed APR dried roots samples (25 mg) and Diol (25 mg) were completely grinded for 3 min by a pestle in an agate mortar. When the sample has been thoroughly disrupted and dispersed, the blend was immediately transferred to a 1 mL empty polypropylene SPE cartridge in which a sieve plate was already putted at the bottom. Then, a second sieve plate was added to the top of the blend and then gently compressed. The elution was performed with 70% methanol-water solution (1 mL) using an aspirator pump. The eluent was centrifuged twice at 14,000 rpm for 10 min prior to UHPLC analysis. Schematic diagram of the Diol-based-MSPD procedure is depicted in Figure 2.

### Ultrasonic Extraction

On the basis of the Chinese Pharmacopoeia Commission (2015), an aliquot of 0.500 g APR sample was accurately weighed and then introduced into a 50 mL stopper conical flask, mixed with 50.00 mL of methanol and weighed the flask. The mixture was extracted ultrasonically (40 kHz, 96% power) for 30 min. After cooling to room temperature, the flask was weighed accurately again and the loss of weight was replenished using with methanol. All the final extract solutions were filtrated through 0.22 μm syringe filters before UHPLC-PDA analysis.

## Results and Discussion

### Optimization of the MSPD Method

#### Type of Sorbent

**FIGURE 3:**
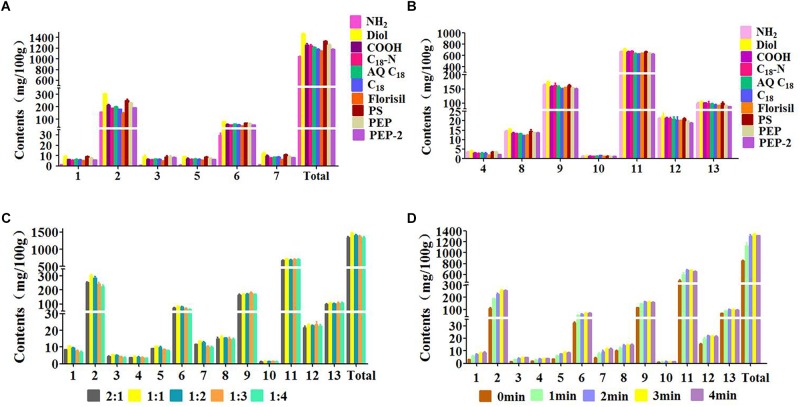
Effects of parameters on efficiency of 13 peaks: (1) neochlorogenic acid, (2) chlorogenic acid, (3) 4-dicafTeoylquinic acid, (4) umbelliferae, (5) isochlorogenic acid B, (6) isochlorogenic acid A, (7) isochlorogenic acid C, (8) columbianetin, (9) columbianetin acetate, (10) imperatorin, (11) osthole, (12) isoimperatorin, (13) columbianadin. **(A,B)** Type of the sorbent, **(C)** Ratio of sample to sorbent, **(D)** Grinding time.

The type of sorbent decided whether produce the high adsorption capacity and selectivity between samples and sorbent. In this study, 10 types of sorbents (C_18_, C_18_-N, AQ C_18_, NH_2_, COOH, Diol, Florisil, PS, PEP, and PEP-2) were investigated. As Figure 3A,B shown, the extraction capacity of Diol was higher than other nine sorbents for all of the target compounds. *T*-test was introduced to evaluate the data, which indicated that there was a significant difference between Diol and other sorbents (*P* < 0.05). The adsorption of the Diol on tested analytes is mainly derived from hydrogen bonding. Meanwhile, the extraction yields of Diol for polar compounds including phenolic acids was high due to the powerful Coulomb field and electrostatic interaction presented inside the cavities of Diol crystals. Furthermore, the carbon chains on the bond phase of Diol provides enough non-polar forces to retain the hydrophobic analytes, such as coumarins. Therefore, Diol was chosen as the optimal sorbent for the subsequent extraction procedure.

#### Mass Ratio of Matrix to Sorbent

It has been reported that the mass ratio of sample to sorbent could affect the interface area between sample and sorbent and then influence the extraction efficiency of target compounds (Rodrigues et al., 2010; Xu et al., 2016). Thus, the mass ratio of sample to sorbent (2:1, 1:1, 1:2, 1:3, 1:4) with the amount of sorbent varying from 12.5 to 100 mg was investigated. It could be seen in Figure 3C, the extraction efficiency of each target compound was distinctly increased when the sample/sorbent ratio increased from 2:1 to 1:1. The reason probably is that the properly amount of sorbent produced the stronger molecular interaction with the sample, such as hydrogen bonding and electrostatic force. With the increasing of the Diol, the extraction efficiency of each target analyte has a slight decrease, which might due to the increasing interaction results in the harder elution process. Therefore, the sample/sorbent ratio of 1:1 was adopted for the following experiments.

#### Grinding Time

The grinding time is also an crucial factor that influences the extraction efficiency (Cao et al., 2016). Enough grinding time not only disperses the sample completely on the solid support, but also promotes the transfer of analytes from sample to dispersing sorbent. To obtain the highest extraction efficiency of the target analytes, different grinding times (0, 1, 2, 3, 4 min) were evaluated. The Figure 3D shows that the extraction efficiency of each target analyte was increased obviously with the increasement of the grinding time (0–3 min). The reason might be the stronger physical and chemical interactions between sample components and Diol. However, the extraction yield exhibited a slight decrease with the grinding time increased from 3 min to 4 min. This phenomenon indicated that overlong grinding time produce too strong adsorption of Diol on target compounds so that the desorption procedure become harder. Thus, a grinding time of 3 min was selected based on the above results.

#### Elution Solvent

The elution solvent has a remarkable influence on effectively eluting the target compounds in the MSPD procedure as a general mobile phase. In this experiment, a series of elution solvents including methanol, ethanol, acetonitrile, propanol and ethyl acetate were selected to investigate. The results indicated that all the target analytes performed the highest extraction efficiency when methanol was chosen as the elution solvent. However, the high polarity analytes were not detected using ethanol, acetonitrile, propanol and ethyl acetate as elution solvent. The reason for this finding probably support by the low polarity of the elution solvent, which was so hard that the high polarity analytes cannot be elute. As a result, methanol was selected as the elution solvent.

**FIGURE 4:**
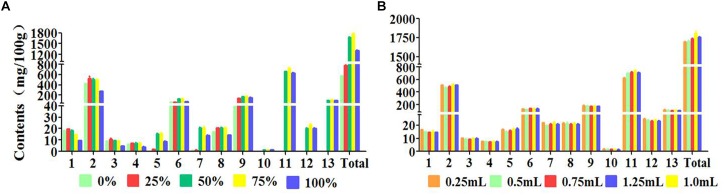
Effects of parameters on efficiency of 13 peaks: (1) neochlorogenic acid, (2) chlorogenic acid, (3) 4-dicaffeoylquinic acid, (4) umbelliferae, (5) isochlorogenic acid B, (6) isochlorogenic acid A, (7) isochlorogenic acid C, (8) columbianetin, (9) columbianetin acetate, (10) imperatorin, (11) osthole, (12) isoimperatorin, (13) columbianadin **(A)** Concentration of elution solvent, **(B)** Volume of elution solvent.

The concentration of elution solvent was another important factor in the step of elution. The elution solvent should have the nearly same polarity with the target compounds in order that the target compounds can be easily eluted by the elution solvent. The results (Figure 4A) shown that the extraction efficiency of the target compounds were increasingly increased with the concentration increased (from water solution to 75% methanol-water solution). The extraction efficiencies of coumarins were unsatisfactory when the concentration increased continuously (from 75% methanol-water solution to 100% methanol). Therefore, 75% methanol-water solution was selected for subsequent experiment.

In order to thoroughly achieve the desorption process using the appropriate volume of eluent, the influence of the eluent volume was also tested. In general, small amount of eluent makes the target compounds eluted incomplete and the target is still adsorbed on the sorbent, which resulted in the lower extraction efficiency. Excessive volume of the eluent may lead to the impurities adsorbed on the sorbent to be eluted at the same time, influencing the analytical detection of the target compounds. The results (Figure 4B) revealed that the extraction recoveries of the target compounds were increased remarkably with the increasement of 75% methanol-water solution volume range from 0.25 to 1.00 mL. However, a slight decrease was appeared when the volume was reached to 1.25 mL, which illustrated that 1.00 mL of 75% methanol-water solution was sufficient to elute 13 target compounds from the samples. On the basis of these results, 1.00 mL 75% methanol-water solution was selected for the following MSPD experiment.

### Method Validation

#### Selectivity and Linearity

The calibration curves (*n* = 8) of 13 analytes were obtained by performing the peak areas as Y-axis and the analyte concentration in g mL^-1^ as X-axis, which ranged from 0.16 to 500 μg mL^-1^. As seen in Table 1, optimal linearities for each analyte with correlation coefficients (*R*^2^) were both higher than 0.9990.

**Table 1:** Linearity, LOD, LOQ and repeatability of the proposed method (*n* = 6).

Compounds	Regressive equation	Linear range (μg/mL)	*R*^2^	LOD (μg/mL)	LOQ (μg/mL)	Repeatability RSD (%)
Neochlorogenic acid	Y = 15148x+245	0.20–50	0.9994	0.10	0.20	4.95
Chlorogenic acid	Y = 18165x+1107	1.00–250	0.9995	0.10	0.20	4.42
4-dicaffeoylquinic acid	Y = 15402x+324	0.16–40	0.9996	0.08	0.16	4.52
Umbelliferae	Y = 34228x+962	0.16–40	0.9994	0.08	0.16	2.47
Isochlorogenic acid B	Y = 14212x+346	0.16–40	0.9996	0.08	0.16	5.01
Isochlorogenic acid A	Y = 26333x-167	0.80–200	0.9998	0.08	0.16	3.45
Isochlorogenic acid C	Y = 20520x+408	0.20–50	0.9998	0.10	0.20	3.94
Columbianetin	Y = 24029x+508	0.20–50	0.9996	0.10	0.20	4.90
Columbianetin acetate	Y = 22452x+3027	0.8–200	0.9990	0.08	0.16	1.61
Imperatorin	Y = 10111x+282	0.16–40	0.9995	0.08	0.16	4.55
Osthole	Y = 23118x+11550	2.00–500	0.9991	0.10	0.20	3.28
Isoimperatorin	Y = 20476x+291	0.20–50	0.9994	0.10	0.20	2.90
Columbianadin	Y = 17976x+17726	0.6–150	0.9995	0.12	0.24	2.95


#### Limits of Detection and Quantification

The Limits of detection (LODs) and limits of quantification (LOQs) were employed to assess the sensitivity of the developed method. The LOD and LOQ of the analytes were calculated as the minimum concentrations on the basic of the signal-to-noise (*S/N*) ratio of 3 and 10, individually. The results are summarized in Table 1, the values of LODs of five compounds ranged from 0.08 to 0.12 μg mL^-1^, while the LOQs ranged from 0.16 to 0.24 μg mL^-1^.

#### Reproducibility and Recovery

The repeatability was evaluated by analyzing the same sample parallelly six times using the developed MSPD method. As summarized in Table 1, the values of relative standard deviations (RSDs) were all less than 5.01%. It was clarified that the developed method had good reproducibility during experiment.

To verify the accuracy of the developed MSPD method, recovery tests were employed to analyze the spiked sample in triplicate. Unspiked samples and spliked samples were simultaneously extracted using the optimum MSPD procedures. The results are listed in Table 2, the mean recoveries of 13 compounds were all in a range of 94.8–107% and the RSDs were all less than 3.22%, which demonstrated that the proposed MSPD method was reliable and effective.

**Table 2:** The results of recovery test (*n* = 6).

Compounds	Original (μg)	Spike (μg)	Detected (μg)	Average recovery (%)	RSD (%)
Neochlorogenic acid	3.72	1.88	5.52	94.8	2.13
Chlorogenic acid	129.01	63.40	196.91	107	1.28
4-dicaffeoylquinic acid	2.22	1.00	3.29	105	1.64
Umbelliferae	2.02	1.02	3.05	98.7	2.53
Isochlorogenic acid B	4.18	2.11	6.31	100	3.10
Isochlorogenic acid A	29.03	11.49	40.84	103	3.22
Isochlorogenic acid C	4.57	2.30	6.90	101	2.46
Columbianetin	5.58	2.68	8.47	107	2.17
Columbianetin acetate	45.33	22.59	68.39	101	2.53
Imperatorin	0.49	0.29	0.77	96.9	2.08
Osthole	187.84	96.84	285.62	100	1.48
Isoimperatorin	5.62	2.76	8.36	99.0	3.20
Columbianadin	27.62	13.68	42.03	98.2	2.51


### Application

To validate the applicability of the traced MSPD method, the developed MSPD method was introduced for the determination of the 13 analytes in six dried roots samples of APR collected from different producing areas under the optimal conditions. The results were summarized in Table 3, The contents of neochlorogenic acid, chlorogenic acid, 4-dicaffeoylquinic acid, umbelliferae, isochlorogenic acid B, isochlorogenic acid A, isochlorogenic acid C, columbianetin, columbianetin acetate, imperatorin, osthole, isoimperatorin and columbianadin in APR were in the range of 0.04–0.15 mg g^-1^, 1.12–5.61 mg g^-1^, 0.03–0.22 mg g^-1^, 0.01–0.24 mg g^-1^, 0.05–0.29 mg g^-1^, 0.03–1.35 mg g^-1^, 0.02–0.22 mg g^-1^, 0.06–0.46 mg g^-1^, 1.09–1.86 mg g^-1^, 0–0.02 mg g^-1^, 3.05–11.17 mg g^-1^, 0.08–0.33 mg g^-1^, 0.96–1.21 mg g^-1^, respectively. It was also observed that osthole was predominant and imperatorin was minimal in the APR.

**Table 3:** Contents of the 13 compounds of APR from 6 batches (*n* = 6).

Content (mg/g)	Production region						
	No. 1 (Hubei)	No. 2 (Hubei)	No. 3 (Sichuan)	No. 4 (Sichuan)	No. 5 (Anhui)	No. 6 (Zhejiang)	No. 6^∗^ (Zhejiang)
Neochlorogenic acid	0.04	0.06	0.14	0.13	0.15	0.15	0.15
Chlorogenic acid	1.12	3.50	5.61	4.97	3.88	5.20	3.95
4-Dicaffeoylquinic acid	0.03	0.04	0.11	0.15	0.22	0.10	0.08
Umbelliferae	0.01	0.01	0.09	0.15	0.24	0.07	0.07
Isochlorogenic acid B	0.05	0.12	0.16	0.20	0.29	0.17	0.13
Isochlorogenic acid A	0.03	0.55	1.35	0.62	0.60	1.35	1.20
Isochlorogenic acid C	0.02	0.08	0.09	0.05	0.06	0.22	0.18
Columbianetin	0.18	0.06	0.15	0.23	0.46	0.22	0.21
Columbianetin acetate	1.09	1.86	1.82	1.66	1.09	1.79	1.75
Imperatorin	–	0.01	0.02	0.02	0.01	0.02	0.02
Osthole	3.05	9.47	11.17	9.31	5.95	7.39	7.08
Isoimperatorin	0.08	0.25	0.30	0.33	0.16	0.24	0.23
Columbianadin	0.96	0.91	1.21	0.81	0.89	1.11	1.07


*^∗^The certain APR sample was extracted by the authoritative extract method (Chinese Pharmacopoeia Commission, 2015).*

Additionally, the No 6 sample was used to evaluate the extraction efficiency of the 13 target analytes using the proposed MSPD method and the traditional UAE method documented from Chinese Pharmacopoeia Commission (2015), respectively. The results were listed in Table 3, shown that there was no obvious difference between the MSPD and UAE method. The results indicated that the effectiveness of MSPD method was nearly as much of the UAE method recorded in Chinese Pharmacopoeia Commission (2015) for extracting APR. Thus, the developed Diol-based-matrix solid-phase dispersion method was applicable for extracting bioactive compounds from the real APR sample.

*Radix Angelicae sinensis* (RAS), named Danggui in Chinese. It has the same species with APR. They belonged to the Angelica L. (Chen et al., 2013). Meanwhile, the main chemical compounds are similar, mostly coumarin and phenolic acid (Yi et al., 2009). As a result, the presented Diol-based-MSPD method might be equally feasible for extracting coumarin and phenolic acid from angelica plant (APR and RAS).

### Comparison of the Proposed Method With Reported Approaches

To assess the usefulness of the developed Diol-based-matrix solid-phase dispersion method, a comparison of the sample amounts, type of solvent, solvent volume, extraction method, extraction time, detection method and detection time of the developed MSPD integrated with UHPLC-PDA method with other reported method including ultrasonic extraction high-performance liquid chromatography (UAE-HPLC), ultrasonic extraction high-performance liquid chromatography coupled with quadrupole time-of-flight tandem mass spectrometry (UAE-HPLC-Q-TOF/MS), and microwave-assisted extraction high-performance liquid chromatography (MAE-HPLC) was performed. The results are shown in Table 4 and it is obviously observed that the other reported methods usually need much time and required a great deal of organic solvents, which generated much environmental insecure waste. However, the developed MSPD method shortened the extraction time, reduced the amounts of sample and decreased the volume of organic solvents. In addition, the MSPD method was in conformity with the principles of green analytical chemistry. Additionally, microwave-assisted extraction was used to extract congeneric plant of APR (RAS), which required a large amount of organic solvent and large amounts of samples. Thus, the proposed MSPD method was a sample, rapid, environment-friendly method for extracting and determining the coumarins and phenolic acids in real APR samples and the related congeneric plant.

**Table 4:** Comparison of the MSPD method with other methods in the determination of compounds in APR.

No	Plant	Extracted compounds	Sample amounts (mg)	Type of solvent	Solvent volume (mL)	Extraction method	Extraction time (min)	Detection method	Detection time (min)	Reference
1	APR	Columbianetin, osthole, isoimperatorin, columbianadin	500	Methanol	20	UAE^a^	20	HPLC-DAD	100	Wang J. R. et al., 2018
2	APR	Columbianetin	100	Methanol	5	UAE^a^	60	HPLC-DAD	18	Zhang et al., 2017
3	APR	Neochlorogenic acid, chlorogenic acid, 4-dicaffeoylquinic acid, umbelliferae, isochlorogenic acid B, isochlorogenic acid A, isochlorogenic acid C, columbianetin, columbianetin acetate, imperatorin, osthole, isoimperatorin, columbianadin	200	70% methanol	10	UAE^a^	20	HPLC-MS	40	Ge et al., 2014
4	RAS	Ferulic acid	1000	90% ethanol	20	MAE^b^	9	HPLC-DAD	18	Liu et al., 2006
5	APR	Neochlorogenic acid, chlorogenic acid, 4-dicaffeoylquinic acid, umbelliferae, isochlorogenic acid B, isochlorogenic acid A, isochlorogenic acid C, columbianetin, columbianetin acetate, imperatorin, osthole, isoimperatorin, columbianadin	25	75% methanol	1	MSPD	3	UPLC-PDA	30	This work


*^*a*^Ultrasonic-assisted extraction.*

*^*b*^Microwave-assisted extraction.*

## Conclusion

A simple and eco-friendly MSPD coupled with UHPLC-PDA method was established and validated for extraction and determination of 6 phenolic acids and 7 coumarins in APR. Diol was employed to extract the target compounds in the application of MSPD. Compared with conventional extraction methods (UAE), the present method is rapid, time-saving and efficient. Briefly, this proposed Diol based MSPD coupled with UHPLC-PDA method could be used for extraction and determination of target compounds from other TCMs.

## Data Availability

All datasets generated for this study are included in the manuscript and/or the supplementary files.

## Author Contributions

Y-XC acquired funding for the research. Y-XC, XG, and JL designed the work. MD and YB performed the experiments. XY and HW analyzed the data. MD and YB wrote the manuscript. Y-XC and XG reviewed the manuscript. All authors discussed the results and approved the final manuscript.

## Conflict of Interest Statement

The authors declare that the research was conducted in the absence of any commercial or financial relationships that could be construed as a potential conflict of interest.

## References

[B1] Barker S. A. Long A. R. Short C. R. (1989). Isolation of drug residues from tissues bysolid phase dispersion. *J. Chromatogr. A.* 475 353–361. 10.1016/S0021-9673(01)89689-8 2777960

[B2] Cao J. Peng L. Q. Xu J. J. (2016). Microcrystalline cellulose based matrix solid phase dispersion microextration for isomeric triterpenoid acids in loquat leaves by ultrahigh-performance liquid chromatography and quadrupole time-of-flight mass spectrometry. *J. Chromatogr. A* 1472 16–26. 10.1016/j.chroma.2016.10.034 27776775

[B3] Cao W. Li C. C. (2018). Herb textual research on and common pairs of doubleteeth pubescent angelica root. *J. Shaanxi Chin. Med. Univ.* 41 127–130.

[B4] Chang Y. X. Zhang Q. H. Li J. Zhang L. Guo X. R. He J. (2013). Simultaneous determination scopoletin, psoralen, bergapten, xanthotoxin, columbianetin acetate, imperatorin, osthole and isoimperatorin in rat plasma by LC-MS/MS for pharmacokinetic studies following oral administration of *Radix Angelicae pubescentis* extract. *J. Pharm. Biomed. Anal.* 77 71–75. 10.1016/j.jpba.2012.12.031 23384552

[B5] Chang Y. X. Zhu Z. W. Li J. Zhang Q. H. Deng Y. R. Kang L. Y. (2011). Quantitative determination of anti-Inflammatory columbianetin in rat plasma by LC-ESI-MS/MS for pharmacokinetic studies after oral administration of duhuo extract. *Chromatographia* 74 639–643. 10.1007/s10337-011-2109-0

[B6] Chen X. P. Li W. Xiao X. F. Zhang L. L. (2013). Phytochemical and pharmacological studies on radix *Angelica sinensis*. *Chin. J. Nat. Med.* 11 0577–0587. 10.3724/SP.J.1009.2013.00577 24345498

[B7] Chen Y. (2014). Research progress of Heracleum’s chemical constituents. *J. Liaoning Chin. Med. Univ.* 16 255–256.

[B8] Chinese Pharmacopoeia Commission (2015). *Pharmacopoeia of the People’s Republic of China*. Beijing: Chinese Pharmacopoeia Commission, 263.

[B9] Enríquez-Gabeiras L. Gallego A. Garcinuño R. M. Fernández-Hernando P. Durand J. S. (2012). Interference-free determination of illegal dyes in sauces and condiments by matrix solid phase dispersion (MSPD) and liquid chromatography (HPLC-DAD). *Food. Chem.* 135 193–198. 10.1016/j.foodchem.2012.04.065

[B10] Ge A. H. Ma W. F. Wang C. P. Li J. Liu E. W. Adelakum T. A. (2014). Ultra high performance liquid chromatography with photodiode array detector and quadrupole time-of-flight tandem mass spectrometry coupled with discriminant analysis to evaluate *Angelicae pubescentis* radix from different regions. *J. Sep. Sci.* 37 2523–2534. 10.1002/jssc.201400289 25044521

[B11] Li J. J. Li Y. Y. Xu D. L. Zhang J. Y. Wang Y. X. Luo C. (2017). Determination of metrafenone in vegetables by matrix solid-phase dispersion and HPLC-UV method. *Food Chem.* 214 77–81. 10.1016/j.foodchem.2016.07.061 27507450

[B12] Liu H. C. Li Q. W. Li S. P. Zou Y. H. Gu A. Y. (2008). The rapid determination of artemisinin by post-column derivatization high-performance liquid chromatography using matrix solid-phase dispersion method. *J. Chromatogr. Sci.* 46 122–126. 10.1093/chromsci/46.2.122 18366870

[B13] Liu J. H. Zschocke S. Reininger E. Bauer R. (1998). Inhibitory effects of angelica pubescens f. biserrata on 5-lipoxygenase and cyclooxygenase. *Planta Med.* 64 525–529. 10.1055/s-2006-957507 9741298

[B14] Liu Y. T. Wang F. Wang G. X. Han J. Wang Y. Wang Y. H. (2010). In vivo anthelmintic activity of crude extracts of Radix *Angelicae pubescentis*, *Fructus bruceae*, *Caulis spatholobi*, *Semen aesculi*, and *Semen pharbitidis* against *Dactylogyrus intermedius* (Monogenea) in goldfish (*Carassius auratus*). *Parasitol. Res.* 106 1233–1239. 10.1007/s00436-010-1799-9 20191290

[B15] Liu Z. L. Wang J. Shen P. N. Wang C. Y. Shen Y. J. (2006) Microwave-assisted extraction and high-speed counter-current chromatography purification of ferulic acid from radix *Angelicae sinensis*. *Sep. Purif. Technol.* 52 18–21. 10.1016/j.seppur.2006.03.009

[B16] Rodrigues S. A. Caldas S. S. Primel E. G. (2010). A simple; efficient and environmentally friendly method for the extraction of pesticides from onion by matrix solid-phase dispersion with liquid chromatography-tandem mass spectrometric detection. *Anal. Chim. Acta* 678 82–89. 10.1016/j.aca.2010.08.026 20869508

[B17] Vela-Soria F. Rodríguez I. Ballesteros O. Zafra-Gómez A. Ballesteros L. Cela R. (2014). Simplified matrix solid phase dispersion procedure for the determination of parabens and benzophenone-ultraviolet filters in human placental tissue samples. *J. Chromatogr. A* 1371 39–47. 10.1016/j.chroma.2014.10.063 25456585

[B18] Wang A. W. Liu Y. Luo Q. Zhai H. Wang F. W. Wang M. (2008). Study on pharmacodynamics of analgesia and anti-inflammation of Duhuo-Jisheng Tang. *Chin. J. Exp. Tradit. Med. Form.* 14 61–64.

[B19] Wang H. L. Jiang Y. Ding M. Y. Li J. Hao J. He J. (2018). Simultaneous determination and qualitative analysis of six types of components in Naoxintong capsule by miniaturized matrix solid-phase dispersion extraction coupled with UHPLC with photodiode array detection and Q-TOF-MS. *J. Sep. Sci.* 41 1897–2104. 10.1002/jssc.201701411 29396922

[B20] Wang J. R. Tan J. Li L. Y. Liu J. L. Zhang J. F. (2018). HPLC fingerprint of *Angelica pubescens* radix and determination of four kinds of coumarin. *Chin. J. Pharm. Anal.* 38 955–963.

[B21] Wang S. C. Sun M. Zhang Y. M. Zhang J. He L. C. (2010) EGFR/cell membrane chromatography-online-high performance liquid chromatography/mass spectrometry method for screening EGFR antagonists from Radix *Angelicae Pubescentis*. *Sci. China Chem.* 53 2357–2362. 10.1007/s11426-010-4010-3

[B22] Xu J. J. Cao J. Peng L. Q. Cao W. Zhu Q. Y. Zhang Q. Y. (2016). Characterization and determination of isomers in plants using trace matrix solid phase dispersion via ultrahigh performance liquid chromatography coupled with an ultraviolet detector and quadrupole time-of-flight tandem mass spectrometry. *J. Chromatogr. A* 1436 64–72. 10.1016/j.chroma.2016.01.046 26830637

[B23] Yang X. W. Liu Y. F. Tao H. Y. Yang Z. Shi S. Y. (2006). GC-MS analysis of essential oils in *Angelicae pubescentis* radix. *China J. Chin. Mater. Med.* 31 663–666. 16830826

[B24] Yi L. Z. Liang Y. Z. Wu H. Yuan D. L. (2009). The analysis of radix *Angelicae sinensis* (Danggui). *J. Chromatogr. A* 1216 1991–2001. 10.1016/j.chroma.2008.07.033 18667208

[B25] Zhang L. Ge Y. Y. Li J. Chang Y. X. (2017). Simultaneous determination of columbianetin-β-D-glucopyranoside and columbianetin by HPLC. *J. Tradit. Chin. Med.* 34 345–348.

